# Spatial–temporal changes in the degradation of marshes over the past 67 years

**DOI:** 10.1038/s41598-022-10104-3

**Published:** 2022-04-12

**Authors:** Jing Tang, Ying Li, Bolin Fu, Xiaomin Jin, Gao Yang, Xing Zhang

**Affiliations:** 1 Northeast Institute of Geography and Agroecology, Chinese Academy of Science, No. 4888 Shengbei Street, Changchun, 130102 Jilin People’s Republic of China; 2 University of Chinese Academy of Sciences, Beijing, 100049 People’s Republic of China; 3 College of Geomatics and Geoinformation, Guilin University of Technology, Guilin, 541004 Guangxi People’s Republic of China

**Keywords:** Environmental sciences, Environmental social sciences

## Abstract

Agricultural reclamation is widely regarded as a primary cause of marshes degradation. However, the process of marshes degradation on different geomorphology has rarely explored, which fail to explain the marshes degradation driven by natural restrictions in detail. The information deficiency unable propounded the targeted suggestions for the sustainable management of marshes. According to the development of China, we quantified the degradation rate of marshes on different geomorphic types from 1954 to 2020 in a typical transect in the Sanjiang Plain. The results indicated that (1) A total of 1633.92 km^2^ of marshes reduced from 1954 to 2020. And 97% (1582.35 km^2^) of marshes were converted to crop cultivation. The process of marshes degradation had obvious historical stages characteristics. The marshes degradation rate showed a trend of increasing first and then decreasing. The most serious period was 1995–2005 (6.29%) which was approximately 35 times of the period of before the reform and opening up (1954–1976) a minimal shrunk period. (2) The background of geological tectonic decided the whole trends in marshes degradation process. The degradation occurred first and worst in the meco-scale recent slow ascent region, and then extended to substantially recent slow subsidence region and the small-amplitude recent slow ascent region. (3) Significant location characteristics of marshes degradation reflected in this research. The spatial location of marshes degradation on the sub-regions sequentially consisted of alluvial plain, lower terrace, high floodplain, micro-knoll, low floodplain, and depressions. (4) Most of the existing marshes of the sub-Sanjiang Plain distribution in the national reserves. This study provides important scientific information for restoration and conservation of marshes.

## Introduction

As the most productive and economically valuable ecosystems^1,2^, Marshes offer multiple ecosystem services, including climate regulation, flood control, carbon storage and so on^3,4^. Simultaneously, they play an important role in biodiversity conservation and regional ecological balance, etc^4,5^. In fact, wetlands contribute directly or indirectly to 75 Sustainable Development Goal indicators of the United Nations Environment Programme (UNEP)^6^. However, with the developing society and the increasing population, wetlands are also suffering unprecedented destruction. For example, China^7,8^, Italy^9^, India^10^, Canada^11^, Brazil^12^ and global monitoring of wetland^13,14^. Therefore, many researchers in different spatial–temporal scale and wetlands types about spatial–temporal characteristic of wetlands change was implement^15–17^. Long-term wetland monitoring studies have shown that the average annual loss rate of wetlands is as high as 50% and human activities are the main cause of wetland degradation, in which agricultural activities are the leading anthropogenic force driving factors^5,13,14^.

However, the degradation process of wetlands is not only driven by human activities but also restricted by the natural environment^18,19^. Studies have shown that geomorphology is an important factor that affects the distribution and genesis of wetland^20–22^. For example, over-humidity or waterlogging on the surface is the key feature to the formation of marshes, the non-zonal nature of which indicates that the hydrological characteristics of a region are limited by geomorphic conditions^23^. Mitchell. M^24^ also stated the importance of the marsh loss to overall estuarine function may depend on the location and type of marsh lost. With the sea-level rise, knowing which marshes are most vulnerable allows for the prioritization of restoration and conservation efforts, minimizing future impacts to estuarine systems. Therefore, understanding the evolution process of wetlands driven by geomorphic restrictions and human activities is of great significance for formulating long-term wetland management strategies and revealing the temporal and spatial characteristics of wetland evolution^25,26^.

China's national conditions determine that China's wetlands degradation has its own national historical characteristic, simultaneously, follows the constraints of nature in space^27^. The Sanjiang Plain is the largest marsh area in China^28^, formed by the alluviation of the Heilongjiang, Nongjiang and Wusuli Rivers. Since 1950s, due to the flat landform and fertile land, now, it has become China’s main commodity grain base with the support of the national reclamation policies^29^. However, the serious shrinkage of marshes in the Sanjiang Plain has caused most of the ecological functions lost^30,31^. Although several ecological conservation policies and management measures were formulated to protect and restore natural ecosystems in this region^16^, the marshes in the Sanjiang Plain has still been reclaimed for farmland constantly^32,33^. So, a comprehensive study focusing on the response of marshes degradation to policies and natural constraints is urgently needed to improve the efficiency of marshes restoration and protection.

The primary objective of this study is to explore the spatial–temporal characteristic of marshes degradation in sub-Sangjiang Plain with geomorphic restrictions and human driven from 1954 to 2020 combine with RS and GIS technology. Specifically, we aimed to (1) exam the landscape pattern changes of sub-Sangjiang Plain in 1954–2020, (2) quantify the degradation of marshes in sub-Sangjiang Plain in different China development historical stage, and (3) explore the degradation of marshes in geographic types. We expect to provide scientific basis and theoretical support for the conservation, restoration and sustainable development of marshes in China.

## Study area

**Figure 1:**
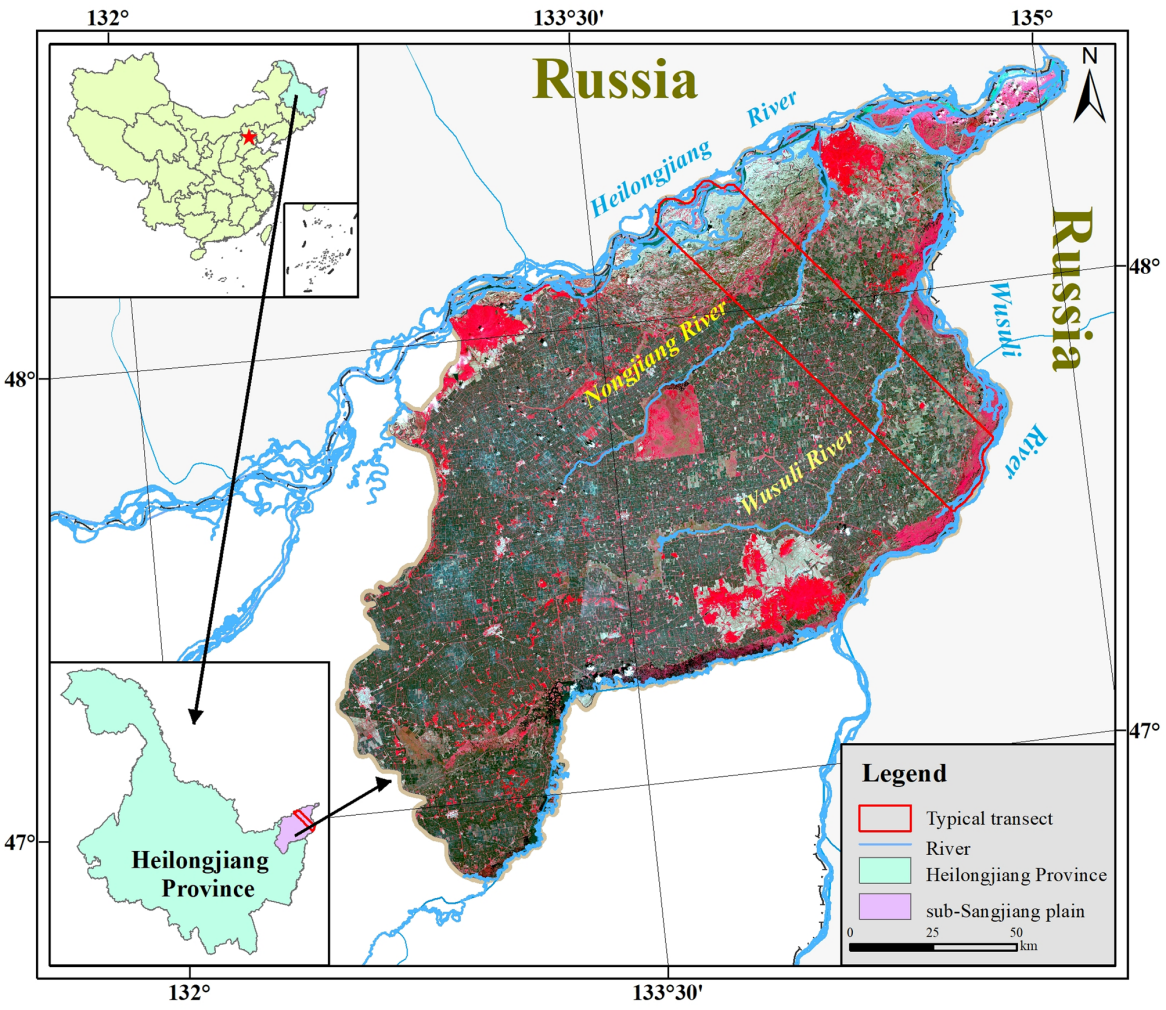
Location of the study area. Figure shows that the sub-Sangjiang Plain is conducted in a subset of Sanjiang Plain, called sub-Sanjiang Plain. This region is the core area of Sanjiang Plain in Heilongjiang Province, northeast China, which locates north of Wanda Mountain.

The study was conducted in a subset of Sanjiang Plain, called sub-Sanjiang Plain. This region is the center area of Sanjiang Plain in Heilongjiang Province, northeast China, which locates north of Wanda Mountain (Fig. 1). The Sanjiang Plain is a low alluvial plain formed by three rivers: Heilongjiang River, Songhua River, and Wusuli River. The total area is 16,073.7 km^2^. The climate is temperate continental with four distinct seasons, including 6 months of freezing conditions^34^. The mean annual precipitation is 603.8 mm and mainly concentrates from June to August. The long freezing periods and clayey soil prevent water from infiltrating to deep layer. The geological structure type is Tongjiang Depression. The low-lying geomorphologic pattern in the area has been shaped by the surface subsidence since the Quaternary. The geomorphology types mainly included riparian zone and terrace. Besides, a small amount of residual hill scatter in the vast low plain and the micro-landform are well developed on the riparian zones. The densely covered river, low and even terrain, and poor drainage rise the water table level in flood season^35,36^. All these natural and geographical conditions promote the formation and development of the marshes. Furthermore, the study area is suitable for the growth of annual crops such as corn, soybean and rice, etc. And the main soil types are meadow soil, swamp soil and albic soils.

## Data preparation and methods

Many previous studies have shown that wetlands change in China have obvious historical characteristics^16,27,37–40^. Reform and opening up is an important turning point of China’s development. China's policy formulation, economic, agricultural and environmental development and changes were closely related to the basic national policy of reform and opening up^41^. Therefore, this study is based on the large social background of reform and opening up. Considering the availability of data and the lag of policy implementation, the land-use/cover datasets (1:100,000) of the typical transect zone in 1954–2020 were chose. The datasets derived from Northeast Institute of Geography and Agroecology (NEIGAE), Chinese Academy of Sciences (CAS) (http://www.iga.ac.cn) and USGS Global Visualization Viewer (https://glovis.usgs.gov/) (Table 1). More detailed time points and reasons were shown in Table 1. In addition, an auxiliary data of 1:500,000 geomorphic map complied by the Institute of Geographic Sciences and Natural Resources Research, Chinese Academy of Sciences (https://www.resdc.cn/data.aspx?DATAID=260).

**Table 1:** Data description of land use data.

Year	Representative time	Data sources	Data acquired
1954	The beginning of new China	NEIGAE,CAS	Land use map of 1954
1976	The beginning of reform and opening up	NEIGAE,CAS	Land use map of 1976
1982	The middle in initial period of reform and opening up	Landsat 1–5 Multispectral Scanner (MSS)image	LM41130271982289HAJ00 LM31220271982177HAJ07 LM31230261982196HAJ07
1985	The beginning of comprehensive reform and opening up period	NEIGAE,CAS	Land use map of 1985
1990	The middle in comprehensive reform and opening up period	Landsat 4–5 Thematic Mapper (TM) image	LT51130271990143BJC00 LT51130271989252BJC00
1995	The beginning of transformation period of reform and opening up	NEIGAE,CAS	Land use map of 1995
2000	The middle in transformation period of reform and opening up	NEIGAE,CAS	Land use map of 2000
2005	The beginning of deepening period of the reform and opening up	NEIGAE,CAS	Land use map of 2005
2010	The middle in deepening period of the reform and opening up	Landsat Thematic Mapper (TM) image	LT51130272010262IKR00 LT51130272011169IKR01
2015	The end of the deepening period of reform and opening up	Landsat 8 OLI (Operational Land Imager) and TIRS (Thermal Infrared Sensor) image	LC81130272015196LGN01 LC81130272015260LGN01
2020	The end of the deepening period of reform and opening up	Landsat 8 OLI (Operational Land Imager) and TIRS (Thermal Infrared Sensor) image	LC81130272020162LGN00 LC81130272020274LGN00

### Data preparation

To meet the purpose of this study, according to the land-use/cover classification system (Table 2) we adjusted the existing achievement land-use/cover data of the NEIGAE, CAS: 1954, 1976, 1985, 1995, 2000 and 2005, firstly. After preprocessing the remote sensing images in 1982, 1990, 2010, 2015 and 2020. Then the corresponding land-use data were obtained with visual interpretation using ArcGIS. The land-use/cover data of 1982 was generated by updating the adjusted land-use/cover maps of 1976 and 1985 with Landsat Multispectral Scanner (MSS) image in 1982.The land use data of 1990 was visual interpreted by the Landsat Thematic Mapper (TM) image of 1990 with the updating to the adjusted land-use/cover maps of 1985 and 1995.The land use data in 2010, 2015 and 2020 were derived from updating adjusted the land-use/cover map of 2005 with the Landsat Thematic Mapper (TM) image and Landsat 8 OLI (Operational Land Imager) and TIRS (Thermal Infrared Sensor) image, respectively. In addition, the overall accuracy of land-use was reached 90.5%.

**Table 2:** Land cover types in 1:100,000 land use data.

Categories	Subcategories
Forest area	Forest land, sparse wooland, shrub woodland
Grassland	High-covered grassland, mid-covered grassland, low-covered grassland
Farmland	Paddy field, dry farmland
Marsh	Freshwater herbaceous wetlands
Water body	Rivers, reservoirs fishery and lakes
Barren land	Lands unused or difficult for using, saline-alkaline land
Build up	Industrial and commercial, residential, transportation ends

### Methods

#### The criterions of transect establishment

The transect is an important method to study the spatial differences, which has been widely used in the fields of geography and ecology^42–44^. The based-transect method has become an important research tool to explore the zonal variation law in International Geosphere-Biosphere Program (IGBP)^43^. In order to accurately explore the temporal-spatial process and characteristics of the marsh loss and degradation in the sub-Sanjiang Plain, the criterions of the establishment of the typical transect are as follows^45^:

1. The transect is essential to cover all geomorphic types of the distribution area of the marshes in the sub-Sanjiang Plain;
2. The transect is essential to cover the main factors of the formation of the marshes;
3. The transect is able to describe the characteristics of the marsh changes in the sub-Sanjiang Plain from 1954 to 2020;
4. The transect region is able to represent the land use patterns of sub-Sanjiang Plain.

#### The condition of typical transect

**Figure 2:**
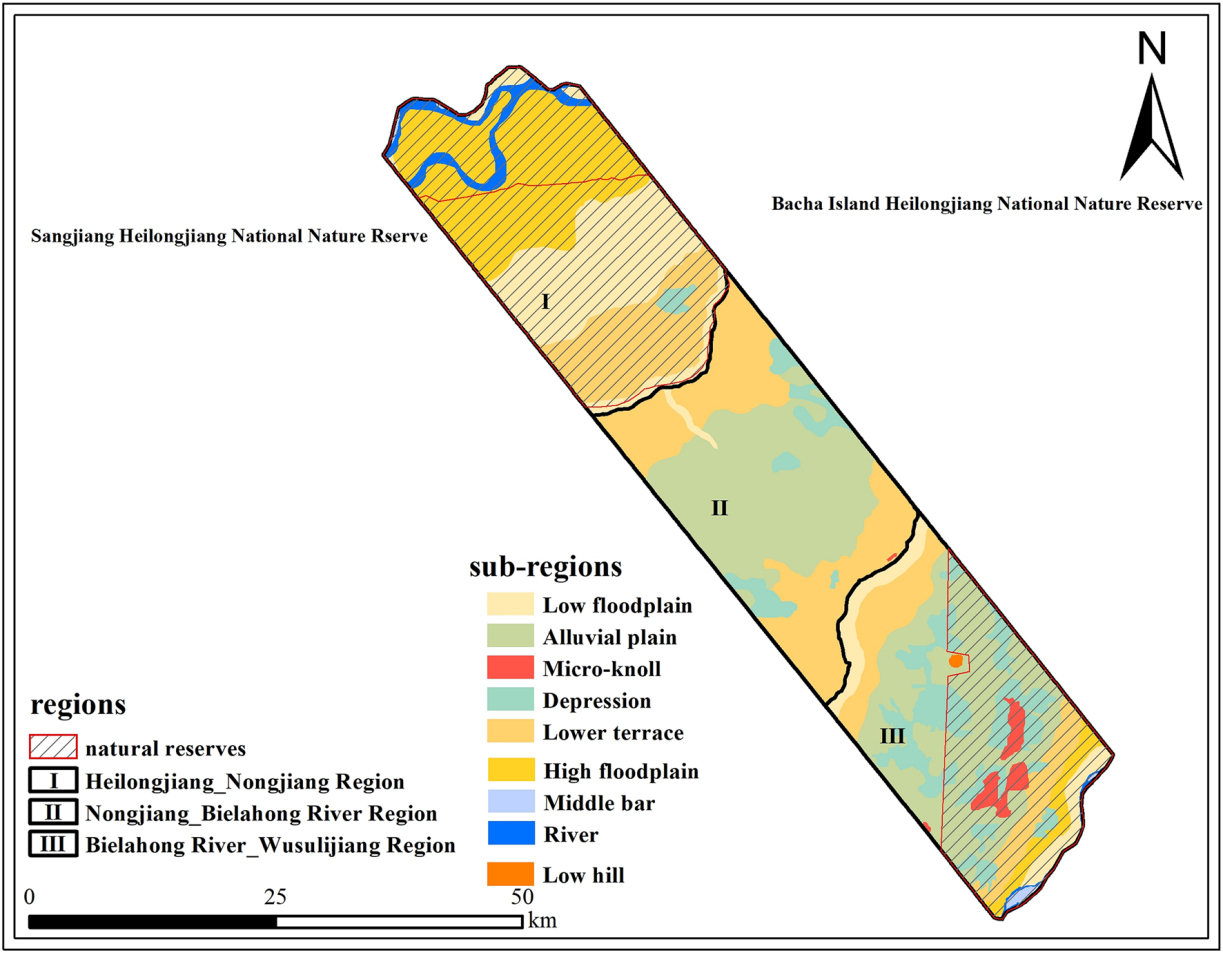
General Situation of the typical transect. Figure shows that the typical transect is divided into three regions named first-region, including Heilongjiang_Nongjiang Region (I), Nongjiang_Bielahong River Region (II) and Bielahong River_Wusuli River Region (III). Then each region is further segmented into some sub-regions with the reference to fluvial geomorphic types, such as river region, middle bar region, depression region, alluvial plain region, micro-knoll region, lower terrace region, low floodplain region, high floodplain region and low hill region.

In this study, we selected a typical transect with the size of 1905 km^2^ from the sub-Sanjiang Plain (Fig. 1). Four main rivers flow through the transect including Heilongjiang River, Nongjiang River, Bielahong River and Wusuli River. The established transect mainly depends on the following factors:

1. The geomorphic types of the transect include alluvial depression, middle bar, alluvial plain, micro-knoll, low floodplain, high floodplain and lower terrace etc. Figure 2, which cover the main geomorphic types of marsh distribution in the sub-Sanjiang Plain;
2. The transect zone is shaped by the Tongjiang Depression. The reasons for the formation of the marsh in the sub-Sanjiang Plain are covered in this transect zone, including low-lying terrain, poor surface drainage and soil water penetration, well developed micro-geomorphy, humid climate and the dense plain river;
3. From 1954 to 2020, the marsh area has decreased by 82% from 1806.7 to 245.7 km^2^, which indicates that there is a severe degradation of marshes;
4. The landscape pattern was changed from the dominated marsh to the dominated farmland during the study period, which is able to represent the changes of land use pattern in the sub-Sanjiang Plain.
5. There are two main nature reserves in the transect zone, which are Sanjiang Heilongjiang National Nature Reserve and Bacha Island Heilongjiang National Nature Reserve (Fig. 2).

#### Segmentation of transect zone

Although the general trend of the neotectonics on sub-Sanjiang plain was descending, it was accompanied by intermittent rising in the process of subsidence, which was mainly controlled by the north-east secondary faults^36^. And due to the surface subsidence since the Quaternary, the geological structure type of the typical transect zone is Tongjiang Depression^20^. The specific manifestations are the Heilongjiang_Nongjiang substantially recent slow subsidence region, the Nongjiang_Bielahong River meco-scale recent slow ascent region and the Bielahong River_Wusuli River small-amplitude recent slow ascent region. Combination of the geological formation movement, the typical transect was divided into three regions named first-region, including Heilongjiang_Nongjiang Region (I), Nongjiang_Bielahong River Region (II) and Bielahong River_Wusuli River Region (III) to reveal the spatial characteristics of marsh degradation. Then each region was further segmented into some sub-regions with the reference to fluvial geomorphic types, such as river region, middle bar region, depression region, alluvial plain region, micro-knoll region, lower terrace region, low floodplain region, high floodplain region and low hill region (Fig. 2).

#### Quantification of wetland degradation

For well understanding the rate of regional marshes changes and their characteristics differences, the marshes dynamic degree was proposed^46^. Wetland dynamic degree can be determined by the land use dynamic degree^47^.

The equation is as follows:

1 $$LC = \frac{{U_{b} - U_{a} }}{{U_{a} \cdot T}} \times 100\%$$

where *U* is the marsh area (km^2^), *b* represents the current year and *a* represents the past years, *LC* is the dynamic degree (annual shrinkage ratio) of marshes area in *T* years.

#### Statistical analysis

In this study we mapped the spatial pattern in typical transect and calculated the conversion among farmland and marshes by ArcGIS software. For a better understanding of the patterns and process in marshes degradation, we quantified both the area and annual shrinkage ratio of marshes (LC) in six stage (Table 3). Specifically, we gathered statistic and compared the spatial–temporal variable changes among the first- regions and sub-regions.

**Table 3:** Description of historical stage.

Stage name	Time intervals
The period of before the reform and opening up	1954–1976
Initial period of reform and opening up	1976–1985
The period of comprehensive reform and opening up	1985–1995
The period of transformation reform and opening up	1995–2005
The period of deepening reform and opening up	2005–2015
The period of deepening reform and opening up	2015–2020

## Results

### Spatial–temporal characteristics of marsh degradation

**Figure 3:**
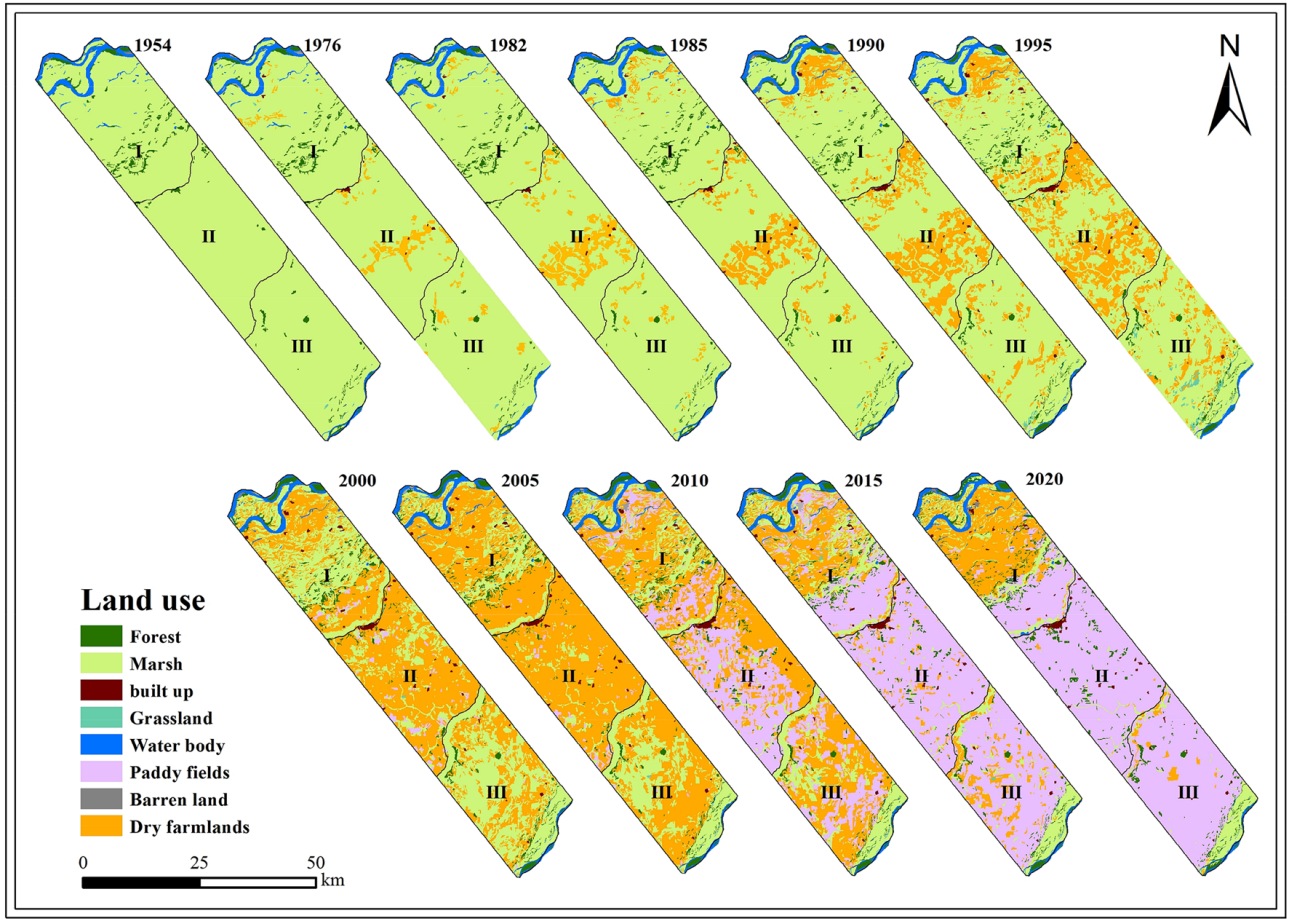
Spatial pattern of land cover types of the typical transect in 1954–2015. Figure shows the changes of landscape patterns in the Sub-Sangjiang Plain from 1954 to 2020. The landscape matrix from marshes changed into farmland.

**Figure 4:**
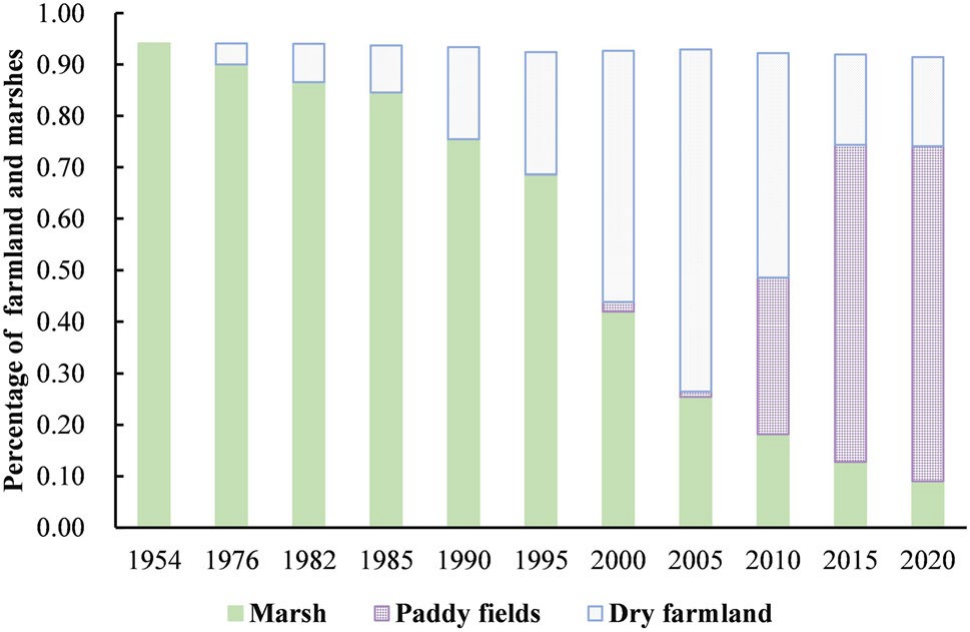
Composition ratio of main land cover types of the typical transect in 1954–2020. Figure shows the percentage of paddy fields, dry farmland and marshes in sub-Sangjiang Plain. And the change of them from 1954 to 2020.

**Figure 5:**
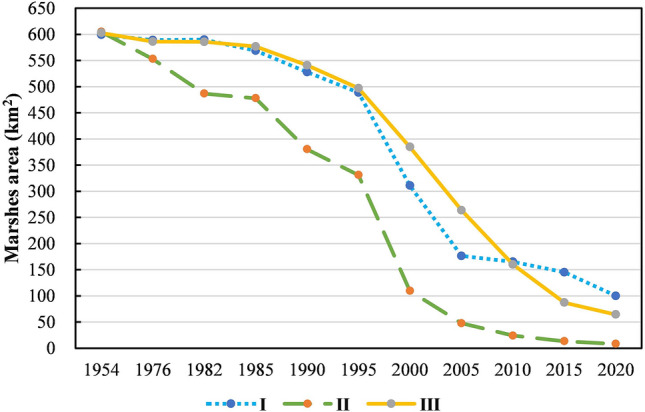
Marshes area the first-region in 1954–2020. Figure shows the area change of marshes in each of the first-region from 1954 to 2020. Which firstly occurred in I region and then expanded to other two regions.

As Fig. 3 shown that the landscape patterns in the typical transect zone have dramatically changed during the 67 years, the landscape matrix from marshes changed into farmland. As Fig. 4 shown that paddy appeared around 2000 and gradually increased eventually, became the main landscape type in sub-Sangjiang Plain. Before this, marshes were only reclaimed into dry farmland. A total of 1633.92 km^2^ of marshes reduced from 1954 (1806.71 km^2^) to 2020 (172.9 km^2^). Meanwhile, there was an increase of 1582.35 km^2^ (82%) of crop cultivation, of which dry farmland and paddy fields were 332km^2^ (17%) and 1250.35 km^2^ (65%) respectively. Additionally, the loss of marshes caused by agricultural encroachment were different in the first-region. Which firstly occurred in I region and then expanded to other two regions (Fig. 5). Compared to region I and III, the marsh degradation rate in region II was faster and worse. In the start of 1954, the areas of marshes in the first-region were 599.18 km^2^, 604.78 km^2^ and 602.70 km^2^ respectively (Fig. 5) but were 100.06 km^2^, 8.36 km^2^ and 64.32 km^2^ in 2020.

Quantification of the marsh degradation was shown in Table 4. The annual shrinkage ratio was continuously increased throughout the whole period which was rose rapidly at a rate of more than 3 times of each history stage in 1954–2005, but started slowly in the period of deepening reform and opening up until 2020. The marsh degradation rate was more notable in the period of transformation reform and opening up stage (1995–2005), which was approximately 35 times of the period of before the reform and opening up (1954–1976) a minimal shrunk period.

**Table 4:** Annual degeneration rate of the marsh in typical transect.

Stage name	Time intervals	Annual shrinkage ratio (LC) (%)
The period of before the reform and opening up	1954–1976	0.2
Initial period of reform and opening up	1976–1985	0.67
The period of comprehensive reform and opening up	1985–1995	1.89
The period of transformation reform and opening up	1995–2005	6.29
The period of deepening reform and opening up	2005–2015	4.96
The period of deepening reform and opening up	2015–2020	5.94

### Dynamic change degree of marsh

The annual degradation rate of marshes in regions I, II and III (Table 5) was all fluctuation, however, the trends of each region has it’s own characteristic. The annual degradation ration of marshes in regions I and II were approximately 4 times and 3 times of the rate respectively from 1954 to 2005, and both were reached the highest value in 1995–2005. However, the rate of region II in each observation stage was both higher than regions I and III. In terms of the highest value, the max annual degradation rate in region II was 8.56%, which was 1.3 and 1.5 times in regions I and III, respectively, although the annual degradation rate decreased in the subsequent period, it was always greater than the highest values of regions I and III; then the annual degradation rate in region I was fall to 1.77% in 2005–2015 which was the minimum of the contemporaneity, and rose to almost the highest rate of region I in the next 5 years. Simultaneously, the annual degradation rate of marsh in region III declined slightly after continue growing from 1954 to 2015. In fact, after the period of the deepening of reform and opening up, the average annual degradation rate has remained high although it has changed.

**Table 5:** The annual variance ratio of the marsh degradation based on the first level division.

Stage name	Time intervals	Annual shrinkage ratio (LC) (%)		
		I	II	III
The period of before the reform and opening up	1954–1976	0.08	0.39	0.13
Initial period of reform and opening up	1976–1985	0.38	1.51	0.18
The period of comprehensive reform and opening up	1985–1995	1.41	3.07	1.38
The period of transformation reform and opening up	1995–2005	6.39	8.56	4.69
The period of deepening reform and opening up	2005–2015	1.77	7.21	6.7
The period of deepening reform and opening up	2015–2020	6.22	7.43	5.24

### Spatial location characteristics of marsh change

#### Heilongjiang_Nongjiang region

**Figure 6:**
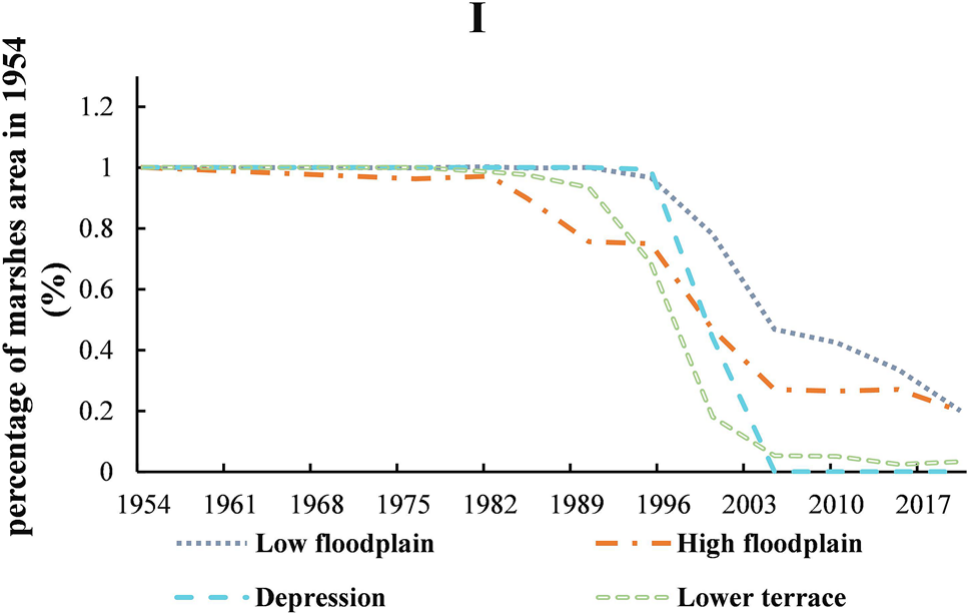
The distribution of marsh during 1954–2020 based on geomorphic types in Heilongjiang_Nongjiang region. Figure shows the marshes in District Heilongjiang_Nongjiang region were mainly distributed on high floodplains, low floodplains, lower terrace, and depression. The degradation of marshes first occurred on the high floodplain, and then appeared in the lower terrace, the floodplain, depressions and low floodplains.

As with Fig. 6 and Table 6 shown, the marshes in District Heilongjiang_Nongjiang region were mainly distributed on high floodplains (202.7333 km^2^), low floodplains (262.992 km^2^), lower terrace (123.456 km^2^), and depression (7.392 km^2^). The sporadic degradation of marshes first occurred on the high floodplain (1954–1976), and then appeared in the lower terrace (1976–1982). When the agriculture reclamation was extended to the floodplain (1990–1995), the marshes on depressions were also began to be continuously cultivated. However, after only 10 years, the marshes on depressions disappeared. It was worth mentioning that before 2005, the mashes were mainly converted into dry land but paddy fields after 2005. The intrusion of agriculture on various geographic was differed in every stage. The periods of most degradation of marshes on the high floodplain from 1985–2005 were reduced by 63%; lower terraces were 90% in 1990–2005; and low floodplains were 77% in 1995–2020. By 2020, the marshes distributed on the lower terraces was less than 0.03 of the original; the high and low floodplains were only 2%.

**Table 6:** The distribution of marsh during 1954–2020 based on geomorphic types in the first level division.

First-region	Sub-region	Percentage of marshes area in 1954 (%)										
		1954	1976	1982	1985	1990	1995	2000	2005	2010	2015	2020
I	Low floodplain	1	1.00	1.00	1.00	1.00	0.97	0.78	0.47	0.42	0.34	0.20
I	High floodplain	1	0.96	0.97	0.90	0.76	0.75	0.47	0.27	0.26	0.27	0.20
I	Depression	1	1.00	1.00	1.00	1.00	0.99	0.44	0.00	0.00	0.00	0.00
I	Lower terrace	1	1.00	0.99	0.98	0.93	0.69	0.18	0.05	0.05	0.02	0.03
I	Middle bar	0	0.00	0.00	0.00	0.00	0.00	0.00	0.00	0.00	0.00	0.00
II	Low floodplain	1	0.86	0.91	0.79	0.56	0.49	0.02	0.02	0.01	0.01	0.00
II	Alluvial plain	1	0.87	0.71	0.71	0.62	0.55	0.15	0.08	0.03	0.02	0.01
II	Depression	1	1.00	0.95	0.95	0.91	0.83	0.53	0.20	0.07	0.02	0.00
II	Micro-knoll	1	1.00	1.00	1.00	0.69	0.69	0.23	0.00	0.00	0.00	0.00
II	Lower terrace	1	0.95	0.89	0.86	0.60	0.50	0.16	0.06	0.04	0.03	0.02
III	Low floodplain	1	1.00	1.00	0.99	0.96	0.97	0.94	0.92	0.89	0.61	0.48
III	High floodplain	1	0.99	0.99	0.98	0.97	0.95	0.93	0.92	0.92	0.92	0.88
III	Alluvial plain	1	0.96	0.96	0.95	0.88	0.79	0.53	0.32	0.13	0.03	0.00
III	Depression	1	1.00	1.00	1.00	0.99	0.98	0.89	0.53	0.24	0.08	0.04
III	Micro-knoll	1	1.00	1.00	0.99	0.79	0.52	0.22	0.02	0.01	0.01	0.00
III	Lower terrace	1	0.93	0.93	0.89	0.81	0.69	0.46	0.31	0.15	0.07	0.03
III	Middle bar	1	1.00	1.04	1.00	0.00	0.00	0.00	0.00	0.00	0.00	0.47
III	Low hill	1	0.63	0.63	0.54	0.54	0.54	0.52	0.34	0.00	0.00	0.00

#### Nongjiang_Bielahong region

**Figure 7:**
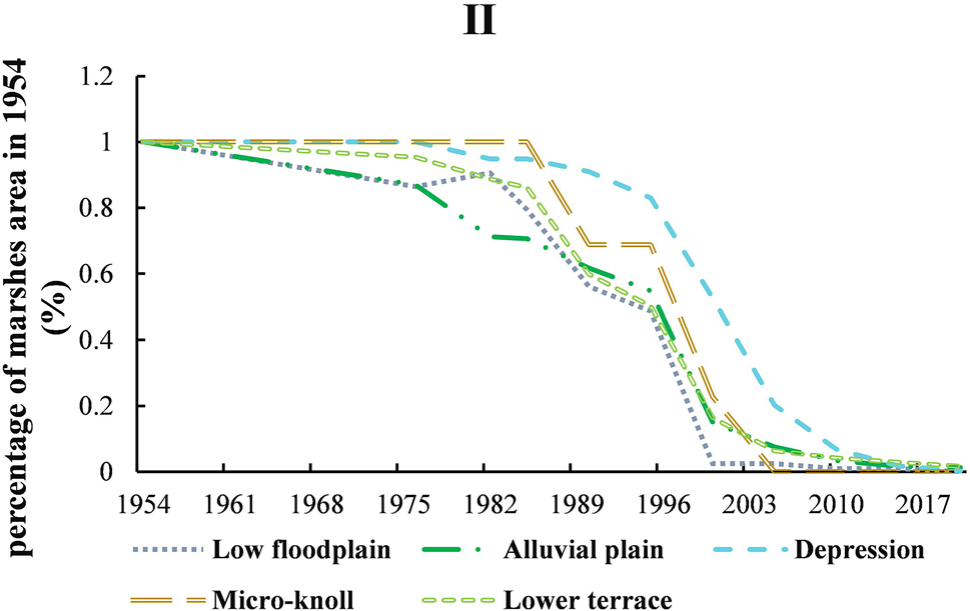
The distribution of marsh during 1954–2020 based on geomorphic types in Nongjiang_Bielahong river. As Figure shown that marshes were mainly distributed on alluvial plains, lower terrace, followed by depressions, and only a few were distributed on low floodplains and few micro-knoll. The reclamation of farmland first started reclamation on alluvial plain and lower terrace, then on low floodplain, followed by depressions, finally micro-knoll.

As Fig. 7 and Table 6 shown that marshes were mainly distributed on alluvial plains (299.916 km^2^), lower terrace (256.839 km^2^), followed by depressions (41.99 km^2^), and only a few were distributed on low floodplains (5.65 km^2^) and few micro-knoll (0.38 km^2^). The reclamation of agriculture on the low floodplain was mainly happened 1982–2000 and the marshes on it were reduced by 89%; alluvial plain were 85% in 1954–2000; lower terraces were 89% in 1976–2005; and depression were 89% in 1990–2005. By 2020, the marshes distributed on the Nongjiang-Bielahong river region were less than 10 km^2^, almostly disappeared. The degradation process of marshes was different among different geographic. The Fig. 7 showed that the reclamation of farmland first started with large-scale reclamation on alluvial plain and lower terrace (1954–1976), then sporadic reclamation on low floodplain (1976–1982), followed by depressions, finally micro-knoll (1985–1990). With the minimum area of marshes (0.378 km^2^), micro-knoll occupied by farmland later but faster than other geographic and completely converted into farmland during 2000–2005. Compared with other geomorphic types, the process of marshes on depression eroded by farmland is the slowest, which was completely occupied by farmland until 2020.

#### Bielahong _Wusuli region

**Figure 8:**
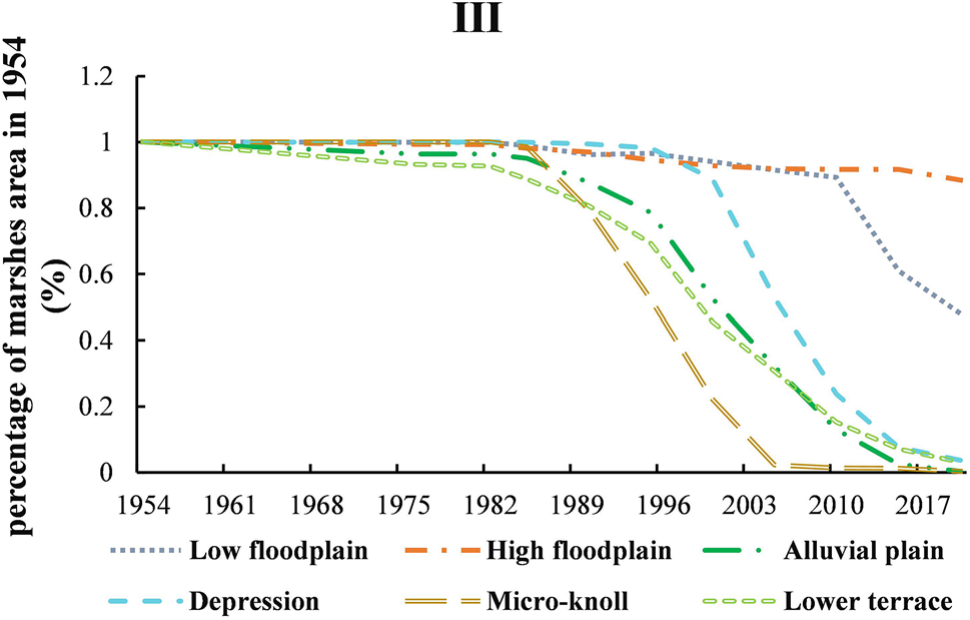
The distribution of marsh during 1954–2020 based on geomorphic types in Bielahong River_Wusuli river. As Figure shown that the main geomorphic types of Bielahong River_Wusuli river region were alluvial plain, depressions, lower terrace, low floodplains, high floodplains, and micro-knoll. Farmland reclamation first occurred in alluvial plain, lower terrace, and high floodplain, then to micro-knoll, low floodplain, and alluvial depression.

The main geomorphic types of Bielahong River_Wusuli river region were alluvial plain, depressions, lower terrace, low floodplains, high floodplains, and micro-knoll. Sporadic farmland reclamation first occurred in alluvial plain, lower terrace, and high floodplain (1954–1976). After 1982, the sporadic reclamation began on micro-knoll and low floodplain which has spread to alluvial depression until 1985. During the entire study period (1954–2020), the marshes on the high floodplain (29.585 km^2^) in wetland reserves were almost uncultivated which only decreased by 12%. Unlike the overall shrinking trend in the region, the marshes distributed on the micro-knoll (24.531 km^2^) were quickly reclaimed after 1985, only 2% of the initial area was left until 2005 and completely disappear in 2020. The ​​marshes distributed on alluvial plain (254.557 km^2^) was the largest which was degraded most seriously. Since 1985, large-scale and rapid farmland opening had occurred on alluvial plain, and the result showed that the marshes in this area were only 2.54 km^2^ in the end. The area of marshes distributed on depressions and were 128.86 km^2^ and 104.460 km^2^, respectively. The time node of lower terraces was basically the same as alluvial plain which was only 3% were retained. The marshes on depression had been reclaimed with highly speed after 2000, only 4% of the initial area were retained in 2020 (Fig. 8, Table 6).

## Discussion

### The historical stage of the change of marshes

**Figure 9:**
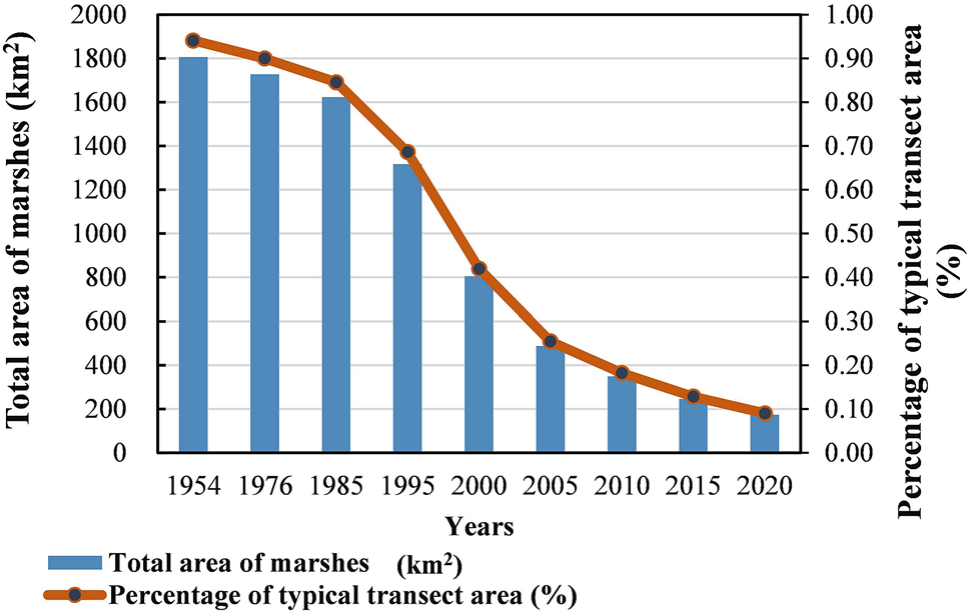
The change of marshes area on typical transect during 1954–2020. Figure shows the total area of wetlands and it’s percentage in sub-Sangjiang Plain is continually decrease from 1954 to 2020.

In the period of before establishment of ‘new’ China, the sub-Sanjiang Plain had just experienced the ban policy of the Qing government, which had the sparsely populated and intact marshes accounting for 94% of the total area of the transect^48^. However, we found that only 0.09% of the total area of marshes in sub-Sangjiang Plain was remain by 2020, the landscape pattern of which had changed completely. Agricultural encroachment caused 86% of the total reduction in marshes in sun-Sangjiang Plain from 1954 to 2020 (Fig. 9). Which was conformity with previous conclusions that agriculture activities are the most main driving cause of marshes^7,27,49^.

Throughout the shrinking process of the marshes in the sub-Sanjiang Plain, although the area of the marshes in the sub-Sanjiang Plain continued to decrease from 1954 to 2020, the whole process had obvious historical stages characteristic (Fig. 3, Table 4). The historical stage characteristics of marshes degradation in the sub-Sanjiang Plain reflect the national government's land policy, demand, and scientific and technological progress after the founding of the People’s Republic of China^29^.

Before 2005, the annual degradation rate of marshes in sub-Sangjiang Plain continued increase. The annual degradation rate of marshes from only 0.2% in the reform and opening up (1954–1976) to 6.29% in the period of transformation reform and opening up (1995–2005), increased 35 times approximately. But which was not a uniform growth and had its own historical characteristics. The “Great Leap Forward” (1958–1960) and “The Down to the Countryside Movement” (1970–1972) activities, made the marshes begun to be reclaimed^27,50^ by a large number of immigrants poured into the Sanjiang Plain. Then the land reclamation demand of “Modern Farm Construction” (1979–1983) and the stimulation of the household responsibility system^51^ both promote the enthusiasm for reclamation. However, due to the limitation of technical conditions the degree of degradation was not serious at begin. With the support of the "Japan Loan Project" many large farming machinery and equipment had been put into use the rate and area of reclamation rose rapidly^52^, the rate of reclamation began to rise rapidly. Why the annual degradation rate in the period of transformation reform and opening up (1995–2005) reached 6.29%, the biggest? That’s because of nearly 40 years of water conservancy project construction, Sangjiang Plain had initially formed three major engineering systems for flood control, waterlogging removal, and irrigation^53^. Good water conservancy and technology conditions provided the greatest convenience for the rapid development of agriculture.

Although the annual degradation rate of marshes was still high, it had begun to decline after 2005. That’s all because of the never slackened protection of wetlands since China joined the "Convention on Wetlands of International Importance, Especially as Waterfowl Habitats" in 1992. In 2002, China implemented the "National Wetland Conservation Plan" (NWCP) and issued two plans for the pre-wetland protection project. In 2014, the red line of the wetland nature reserve was delineated. A series of policies about protection wetlands achieved significant effects in the protection and restoration, especially the wetlands in the Sanjiang Plain^54^. in addition, a national plan to adjust agricultural plantation structures was promoted and implemented actively by the local government, large areas of dry land converted into higher economic benefits paddy fields, reducing the reclamation of marshes. However, the policies of direct agricultural subsidy on crops after 2004 and cancelling taxes on crops from 2006 might have also encouraged farmers to convert marshes to cropland to get more income^38,55,56^. Additionally, the establishment of the World Trade Organization (WTO) in 2001 expanded the grain trading market which also made grain prices rose^16^. Then serious illegal cultivation has occurred. In a word, the drive of benefits caused the loss of marshes and the continuous increase of farmland.

### The spatial–temporal characteristics of the change of marshes

The statistical results of the first regions and sub-regions in geomorphology indicated that the degradation process of marshes in the sub-Sanjiang Plain showed obvious spatial location differences in different historical stages and the degree of reclaiming of marshes on different geomorphic was different. From 1954 to 2020, because of the geological tectonic, the marshes degradation first occurred in the meco-scale recent slow ascent region which was most serious region, and then expanded to the substantially recent slow subsidence region and the small-amplitude recent slow ascent region. It was worth to note that the marshes degradation in the small-amplitude recent slow ascent region was still a trend to continue to expand. In different geomorphic regions, the degradation of marshes first began in alluvial plain, gradually spread to lower terrace and high floodplain, then followed to micro-knoll and low floodplain, and finally extended to depressions. This result fully reflected the restriction on farmland reclamation by geomorphic conditions, reflecting the evolution of the man-land interrelations in the sub-Sanjiang Plain during 1954–2020.

Before the period of comprehensive reform and opening up (1985–1995), marshes reclamation mainly relied on manpower and nature conditions while being restricted by nature in the period of relative backward time which lack of machinery and equipment. Therefore, the reclamation of farmland first occurred in places with superior natural conditions, convenient transportation and easy access, and high population density. And the expansion of reclamation was centered on newly-built residential areas^55^.

As Figs. 3 and 5 shows that both region I and III were sporadic reclamation, continuous reclamation only in region II, and all occurred on the more accessible geomorphic alluvial plains, lower terrace and floodplains (Table 6). Since then, the hydrological cycle of marshes had been changed because of the technology and productivity improved, reconstruction of wells, channels and well irrigation marshes project and continuous farmland reclamation which caused the edges of marshes continue to retreat to the depths of the marshes, and the boundaries of farmland continued to invaded. Conversions between marshes and farmland set up a positive feedback system in the marshes which transforms the marshes into upland, suitable for agricultural operations, and increase of agricultural operations further transforms parts of the marshes^25^. So far, human beings have moved from the stage of being greatly restricted by nature into a process in which humans can vigorously transform nature.

The reclamation sites of marshes had expanded from relatively flat alluvial plains and lower terrace to unsuitable micro-knoll and depressions. The distribution of farmland has also begun to deviate from residential areas and expand linearly along ditches and rivers. In the period of deepening reform and opening up (2005–2020), due to the insufficient reserve resources and implementing national protective measures, the degradation process of marshes had been suppressed to a certain extent, then the man-land interrelations in the sub-Sanjiang Plain had begun to develop in a good direction.

### Implications of natural wetland management

The degradation of marshes in sub-Sanjiang Plain had been controlled to a certain extent, however, the marshes in sub-Sanjiang Plain still urgently need targeted management measures to promote the sustainable development of marshes.

Our study found that most of the existing wetlands in the sub-Sanjiang Plain exist in the National Nature Reserve. Which shows that the establishment of nature reserves plays an important role in the protection of wetlands. Therefore, the conservation and rehabilitation of natural wetlands needs consistent attention from both the government and the public. First, increasing the effectiveness protected areas of wetlands are essential measures for wetlands management. Second, improve the supervision of government to decline the phenomenon of illegal reclamation. Third, a sound national regulatory law is needed to solve the problem of varied protection level in natural wetland conservations several caused by government departments jointly controlled.

Research results proved that the degradation degree and sequence of marshes distributed on different geomorphic types are different. So, we reasonably inferred that the wetlands restoration speed and ultimate degree of restoration with different geomorphological backgrounds will also be limited by its natural conditions. Therefore, the priority areas for restoration should thus focus on the wetlands lost recently such as such as degraded wetlands isolated from large areas (depressions and mcro-knoll), or areas surrounding important wetlands for biodiversity conservation and ecosystem services. As for areas that have been cultivated for a long time such as alluvial plains, considering the changes in food security and hydrological conditions, dry farmland can be transformed to green paddy fields to create artificial wetlands. Improving the living environment quality of waterfowl.

It is impossible to separate the wetlands from agriculture on the Sanjiang Plain. Consider only the protection of wetlands without considering the development of agriculture is unadvisable^57^. The serious problems faced by wetlands are not only direct erosion, but also the opposition between food security and wetland protection, water use conflict between wetland and agriculture, and the pollution caused by the intensive application of chemical fertilizers and pesticides^58,59^. A balanced model that maximizes the benefits of agricultural production and life while ensuring the sustainability of wetlands needs to be explored. For example, develop green leisure agriculture to reduce chemical pollution; change the flood irrigation method of paddy field to centralized irrigation to reduce the conflict between paddy field irrigation and wetland water use.

## Conclusion

According to the historical phases, this study adopted the time series of marsh dynamic change datasets derived from the existing land use data and Landsat series remote sensing images which revealed the changes of land-use/cover patterns in the sub-sanjiang Plain. Study showed that the main reason for the shrinkage of the marshes was the occupation of farmland and the process of marshes farming is the evolution process man-land interrelations in the sub-sanjiang Plain. The process of marshes farming in the sub-sanjiang Plain exhibited significant historical stages and location characteristics with the development of the China and policy orientation was an important factor in the transformation of the human-land interrelations.

The period of transformation reform and opening up (1995–2005) was the most severe period of marshes degradation in the sub-sanjiang Plain which was also the most intense period of human-land conflict. After 2005, the degradation of marshes was prevented in a certain extent due to various protection policies, measures, and the establishment of natural reserves of wetlands. Most of the existing wetlands are distributed in national natural reserves. The marsh degradation was determined by the general regional geomorphic types.

During the study period, the degradation first occurred in the meco-scale recent slow ascent region, and then extended to the substantially recent slow subsidence region and the small-amplitude recent slow ascent region. The process of marshes degradation sequentially consisted of firstly began in alluvial plain, gradually spread to lower terrace and high floodplain, then followed to micro-knoll and low floodplain, and finally extended to depressions.

## Data Availability

The datasets used during the current study are available from the corresponding author on reasonable request.
